# Emergence of Mobilized Tigecycline Resistance Gene Cluster *tmexCD1-toprJ1* in *Raoultella ornithinolytica* From a Swine Farm, China

**DOI:** 10.1155/tbed/6690944

**Published:** 2025-12-18

**Authors:** Hanyun Wang, Yujing Zhang, Zhenyu Wang, Yawen Xu, Le Zhou, Jing Wang, Xinan Jiao, Lin Sun

**Affiliations:** ^1^ Jiangsu Key Laboratory of Zoonosis, Yangzhou University, Yangzhou, China, yzu.edu.cn; ^2^ Jiangsu Co-Innovation Center for Prevention and Control of Important Animal Infectious Diseases and Zoonoses, Yangzhou University, Yangzhou, China, yzu.edu.cn; ^3^ Department of Clinical Laboratory, Yangzhou Center for Disease Control and Prevention, Yangzhou, China; ^4^ Institute of Medical Sciences, School of Public Health, Xinjiang Medical University, Urumqi, China, xjmu.edu.cn; ^5^ Key Laboratory of Prevention and Control of Biological Hazard Factors (Animal Origin) for Agrifood Safety and Quality, Ministry of Agriculture and Rural Affairs of China, Yangzhou University, Yangzhou, China, yzu.edu.cn

**Keywords:** IS26, *Raoultella ornithinolytica*, swine farm, tigecycline resistance, *tmexCD-toprJ*

## Abstract

Tigecycline is considered a last‐line therapeutic option for treating infections caused by multidrug‐resistant (MDR) Gram‐negative bacteria. The emergence of *tmexCD1-toprJ1*, a plasmid‐mediated resistance‐nodulation‐division (RND) efflux pump gene cluster, poses a growing threat to tigecycline efficacy. While this gene cluster has primarily been identified in *Klebsiella pneumoniae*, its presence in other species remains poorly characterized. In this study, we investigated the occurrence, genetic features, resistance profile, and virulence potential of *tmexCD-toprJ*‐positive *Raoultella ornithinolytica* isolates from a swine farm in China. A total of 126 samples were collected from a swine farm and screened for tigecycline‐resistant isolates. Species identification was performed using MALDI‐TOF MS, while PCR and sequencing were applied to detect *tmexCD-toprJ*. Antimicrobial susceptibility testing was assessed by agar and broth microdilution methods. Whole‐genome sequencing with hybrid assembly provided insights into genetic organization. Conjugation and electroporation experiments were conducted to assess plasmid mobility, and virulence was assessed using the *G. mellonella* infection model. Six *R. ornithinolytica* isolates (4.76%) were identified as carrying *tmexCD1-toprJ1* and exhibited multidrug resistance, including reduced susceptibility to tigecycline (minimal inhibitory concentrations [MICs] 4–16 mg/L). The resistance genes were located on highly similar IncFIB plasmids, which lacked typical conjugative elements. However, IS26 sequences flanking the gene cluster suggested potential for horizontal transfer. Phylogenetic analysis indicated possible clonal dissemination. All isolates carried chromosomally encoded virulence genes, and in vivo assays in *G. mellonella* revealed moderate to high pathogenicity. These findings expand the ecological distribution of *tmexCD1-toprJ1* to *R. ornithinolytica* in livestock environments, underscoring the role of swine farm water systems as potential reservoirs. The coexistence of antimicrobial resistance and virulence determinants highlights the urgent need for strengthened surveillance and containment strategies to prevent further dissemination of tigecycline resistance.

## 1. Introduction

Tigecycline is a broad‐spectrum glycylcycline antibiotic and a last‐resort treatment against multidrug‐resistant (MDR) Gram‐negative pathogens [1]. However, the rising prevalence of tigecycline‐resistant Enterobacteriaceae has compromised its clinical efficacy and threatens global public health [2–5]. Among the resistance mechanisms, the plasmid‐mediated resistance‐nodulation‐division (RND)‐type efflux pump gene cluster *tmexCD-toprJ* has recently emerged as a major contributor [6]. First reported in *Klebsiella pneumoniae*, this cluster has also been identified in *Proteus* spp. and *Aeromonas* spp., indicating its potential for horizontal gene transfer and broad dissemination [7–11].

*Raoultella ornithinolytica* is a Gram‐negative aerobic bacillus within the Enterobacteriaceae family, reclassified from *Klebsiella* spp. based on 16S *rRNA* and *rpoB* gene phylogeny [12, 13]. Although primarily associated with soil and water [14–16], it has increasingly been recognized as an opportunistic pathogen causing urinary tract infections, respiratory tract infections, and bacteremia [15, 17–20]. Recent studies have reported MDR *R. ornithinolytica* strains harboring extended‐spectrum *β*‐lactamase (ESBL) genes (*bla*_CTX-M−3_, *bla*_CTX-M−15_), carbapenemase genes (*bla*_NDM−1_, *bla*_KPC−2_, *bla*_IMP−4_, *bla*_OXA−48_), mobile colistin resistance genes (*mcr-1*, *mcr-8*) and tigecycline resistance genes such as *tet*(X4) and *tmexCD2-toprJ2*. These resistant strains have been isolated from humans, animals, plants, and the environment, demonstrating the species’ ecological adaptability and its emerging role as a reservoir of antimicrobial resistance genes [11, 21–29]. The pathogenic potential of *Raoultella* spp. complicates clinical management and highlights the need for surveillance [30, 31].

China is the world’s largest producer and consumer of live pigs [32], and intensive pig farming has rapidly expanded to meet rising demand for pork. High‐density animal production systems increase the risk of disease transmissions [33]. Since the 1950s, antibiotics, particularly tetracyclines, have been widely applied in livestock for disease prevention, growth promotion, and feed efficiency [32, 34, 35]. However, long‐term and excessive antibiotics use has accelerated the emergence and dissemination of resistant bacteria and genes [36–39]. A large proportion of these drugs is excreted unmetabolized, imposing selective pressure on environmental microbial communities, and promoting the persistence and transfer of resistance determinants [40, 41]. As a result, pig farm environments have become major reservoirs of multidrug resistance genes, facilitating their spread within farm and beyond [31, 42–44]. More importantly, resistant bacteria and antibiotic resistance genes (ARGs) may enter the food chain, and accumulate in humans, posing a direct threat to public health [45, 46].

Against this background, the present study investigated the antimicrobial resistance profiles, genomic characteristics and horizontal transfer potential of *tmexCD1-toprJ1*‐positive *R. ornithinolytica* isolates recovered from a swine farm in China. The findings provide new insights into the dissemination of this emerging resistance determinant and its implications for animal and human health.

## 2. Materials and Methods

### 2.1. Sample Collection and Bacterial Isolation

In May 2022, a total of 126 samples were collected from a swine farm in Jiangsu Province, China. Detailed sample information is provided in Supporting Informations 1: Table S1. Samples were obtained using sterile cotton swabs moistened with sterilized saline and transferred into 5 mL sterile centrifugal tubes containing Luria–Bertani (LB) broth. After 16 h of enrichment at 37°C, the cultures were streaked onto MacConkey inositol adonisol carboxy penicillin agar (Haibo, Qingdao, China) supplemented with 2 mg/L tigecycline to selectively isolate tigecycline‐resistant strains. One colony per plate was picked and subjected to three successive rounds of purification. Bacterial species were identified using matrix‐assisted laser desorption/ionization‐time of flight mass spectrometry (MALDI‐TOF MS, Bruker, Bremen, Germany). PCR screening for the *tmexCD-toprJ* gene cluster was performed using previously described primers and protocols.^6^

### 2.2. Antimicrobial Susceptibility Testing

Minimal inhibitory concentrations (MICs) were determined by agar dilution for most antibiotics, with broth microdilution applied specifically for tigecycline and colistin. The antimicrobial agents tested included ampicillin, cefotaxime, meropenem, streptomycin, gentamicin, amikacin, tetracycline, tigecycline, chloramphenicol, florfenicol, nalidixic acid, ciprofloxacin, trimethoprim‐sulfamethoxazole, fosfomycin, and colistin. Results were interpreted according to the Clinical and Laboratory Standards Institute (CLSI) guidelines (M100‐S32, 2022) [47], except for streptomycin, tigecycline, and florfenicol, which were interpreted using the European Committee on Antimicrobial Susceptibility Testing (EUCAST) criteria (https://www.eucast.org/). *Escherichia coli* ATCC 25922 served as the quality control strain. Antimicrobial susceptibility testing was performed in three independent biological replicates.

### 2.3. Plasmid Transfer Experiments

The transferability of the *tmexCD-toprJ* gene cluster was evaluated via conjugation assays using *E. coli* C600 (streptomycin‐resistant) as the recipient strain. Donor and recipient cells were mixed at a 1:4 ratio and co‐incubated at 37°C for 24 h. The resulting mixture was then plated onto MacConkey agar supplemented with 2 mg/L tigecycline and 3000 mg/L streptomycin, followed by overnight incubation at 37°C.

In parallel, plasmid transferability was also assessed by electroporation into *E. coli* DH5*α*. After transferability, transformants were recovered in LB broth at 37°C for 1 h and selected on LB agar plates containing 2 mg/L tigecycline. Presence of the *tmexCD-toprJ* gene cluster in transconjugants and transformants was confirmed by PCR, and their antimicrobial susceptibility profiles were determined as described above. The mobility of the plasmids was further analyzed using oriTDB (https://bioinfo-mml.sjtu.edu.cn/oriTDB2/) [48].

### 2.4. *Galleria mellonella* Infection Model

The virulence potential of *tmexCD-toprJ*‐positive isolates was evaluated using the *G. mellonella* larvae infection model, with minor modifications to previously described protocols [30]. Overnight bacterial cultures were washed with phosphate‐buffered saline (PBS) and adjusted to 1 × 10^6^ CFU/mL. Ten larvae were randomly assigned to each group and injected in the right hind proleg with 10 *μ* L of bacterial suspension. Larvae injected with 10 *μ* L of PBS served as the negative control.

The hypervirulent *K. pneumoniae* strain H27 and low‐virulence strain L51 were included as high‐ and low‐virulence reference strains, respectively, to provide comparative benchmarks for virulence assessment [49]. Infected larvae were incubated at 37°C in the dark, and survival was monitored at regular intervals over a 72 h period. Each experiment was performed independently three times, and the survival curves shown represents data from one representative experiment, as similar trends were observed across replicates. Larvae that failed to respond to gentle probing were considered dead.

### 2.5. Whole Genome Sequencing and Bioinformatic Analysis

Genomic DNA was extracted using the TIANamp Bacteria DNA extraction kit (DP302‐02, Tiangen Biotech, Beijing, China). Short‐read sequencing was performed on the Illumina NovaSeq 6000 platform (Illumina, San Diego, CA, USA), and long‐read sequencing was conducted on the Oxford Nanopore platform (Oxford Nanopore Technologies, Oxford, UK). Hybrid de novo assembly was achieved using SPAdes 3.11 [50] and Unicycler 0.4.9 [51], followed by error correction with Pilon 1.23 [52]. Genome annotation was conducted with Prokka 1.13 [53].

Insertion sequences (ISs) and transposons were identified using the ISFinder [54]. Antimicrobial resistance genes, and multilocus sequence typing (MLST) were analyzed employing Kleborate 2.0 [55]. Virulence‐associated genes were identified using the Virulence Factor Database (VFDB, http://www.mgc.ac.cn/VFs, local offline copy, downloaded in January 2024). Plasmid replicon types were identified using PlasmidFinder 2.1 (https://cge.food.dtu.dk/services/PlasmidFinder/). Comparative plasmid visualization was performed using the BLAST Ring Image Generator (BRIG) [56].The genetic environment surrounding the *tmexCD-toprJ* gene cluster was visualized using Easyfig 2.2.5 [57]. A phylogenetic tree based on core‐genome single nucleotide polymorphisms (SNPs) was generated using ParSNP [58]and visualized with the chiplot web tool (https://www.chiplot.online/) [59].

## 3. Results

### 3.1. Prevalence of *tmexCD1-toprJ1* in Swine‐Associated *R. ornithinolytica* Isolates and Antimicrobial Susceptibility

Out of 126 swine‐associated samples screened on MacConkey inositol adonisol carboxy penicillin agar supplemented with 2 mg/L tigecycline, six tigecycline‐resistant isolates (4.8%) were obtained (Supporting Informations 1: Table S1). PCR amplification and subsequent Sanger sequencing, confirmed the presence of the *tmexCD1-toprJ1* gene cluster in all six isolates. Species identification using MALDI‐TOF MS further revealed that all isolates were *R. ornithinolytica*.

All *tmexCD1-toprJ1*‐positive isolates exhibited multidrug resistance, with tigecycline MICs ranging from 4 to 16 mg/L. These isolates demonstrated resistance to multiple antibiotics, including ampicillin, cefazolin, streptomycin, gentamicin, tetracycline, chloramphenicol, florfenicol, nalidixic acid, ciprofloxacin, sulfamethoxazole/trimethoprim, and colistin. However, they remained susceptible to meropenem, amikacin, and fosfomycin (Table 1).

**Table 1:** The minimum inhibitory concentrations of tested antimicrobialagents for the studied bacterial isolates.

Strains	MIC (mg/liter)														
	AMP	CTX	MEM	STR	GEN	AMK	TET	TIL	CHL	FFC	NAL	CIP	SXT	FOS	CL
GY22PK011	>**128**	**32**	0.06	>**256**	>**128**	4	>**128**	**8**	**128**	**128**	>**256**	**16**	**64**	64	**16**
GY22PK019	>**128**	**32**	0.06	>**256**	>**128**	4	>**128**	**4**	**128**	**128**	>**256**	**16**	**64**	64	**16**
GY22PK020	>**128**	**32**	0.06	>**256**	>**128**	4	>**128**	**8**	>**128**	**128**	>**256**	**16**	**64**	64	**16**
GY22PK021	>**128**	**32**	0.06	>**256**	>**128**	4	>**128**	**8**	**128**	**128**	>**256**	**16**	**64**	64	**16**
GY22PK022	>**128**	**16**	0.06	>**256**	>**128**	4	>**128**	**16**	**128**	**128**	>**256**	**16**	**64**	64	**16**
GY22PK023	>**128**	**8**	0.06	**256**	**32**	4	>**128**	**8**	**128**	**128**	>**256**	**16**	**64**	64	**8**

*Note:* MICs of tigecycline and colistin were determined by microdilution andthose of other antibiotics by agar dilution. Bold numbers indicate that the isolates are resistant to the tested antimicrobial agent. AMK, amikacin; AMP, ampicillin; CHL, chloramphenicol; CIP, ciprofloxacin; CL, colistin; FFC, florfenicol; FOS, fosfomycin; GEN, gentamicin; MEM, meropenem; NAL, nalidixic acid; STR, streptomycin; SXT, sulfamethoxazole/trimethoprim; TET, tetracycline; TIL, tigecycline.

### 3.2. Genomic Characteristics

To investigate the genomic features of the *tmexCD1-toprJ1*‐positive isolates, whole‐genome sequencing was performed. Hybrid assembly revealed that strain GY22PK011 harbored one chromosome and a single plasmid, whereas the remaining five isolates each contained three plasmids. All chromosomes encoded a class A *β*‐lactamase gene *bla*_PLA1a_, and the fosfomycin resistance gene *fosA*. Plasmid replicon typing indicated that the *tmexCD1-toprJ1*‐carrying plasmids belonged to the IncFIB incompatibility group.

**Figure 1:**
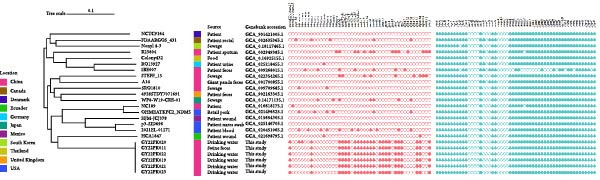
Phylogenetic relationships and distribution of *tmexCD1-toprJ1*‐carrying *R. ornithinolytica* isolates. Maximum‐likelihood phylogeny based on core‐genome single‐nucleotide polymorphisms (SNPs) of 24 *R. ornithinolytica* isolates from diverse geographical locations. Colored boxes on the left indicate the country of origin (see legend). The sources of isolation and corresponding GenBank accession numbers are listed beside the tree. The presence (filled circles) or absence (open circles) of major antimicrobial resistance and virulence genes is shown on the right. Six isolates from this study (GY22PK011‐GY22PK023) were derived from livestock‐associated drinking water and swine‐feces samples. The scale bar represents nucleotide substitutions per site.

In addition to *tmexCD1-toprJ1*, these plasmids carried a diverse array of resistance genes associated with multiple antimicrobial classes. These included the *β*‐lactamase gene *bla*_DHA−1_, aminoglycoside resistance genes such as *aph*(*4*)*-Ia*, *aac*(*3*)*-IV*, *aadA1 aadA2 strA* and *strB*, the quinolone resistance gene *qnrB4*, the chloramphenicol resistance gene *cmlA1*, and two sulfonamide resistance genes, *sul1* and *sul3* (Table 2, Figure 1). The co‐occurrence of these resistance determinants on *tmexCD1-toprJ1*‐bearing plasmids highlights the potential of *R. ornithinolytica* to act as a reservoir and vector for the horizontal transmission of multidrug resistance elements.

**Table 2:** Whole genomes of *tmexCD1-toprJ1* ‐positive *Raoultella ornithinolytica* isolates in this study.

Isolate/replicon	Size (bp)	Resistance genes	Plasmid replicon
GY22PK011			
Chromosome	5,562,654	*bla*_PLA1a_, *fosA*	—
pGY22PK011_1	167,402	*tmexCD1*‐*toprJ1*, *bla*_DHA-1_, *aph(4)-Ia*, *aac(3)-IV*, *aadA1*, *aadA2*, *strA*/*B*, *qnrB4*, *cmlA1*, *sul1*, *sul3*	IncFIB
GY22PK019			
Chromosome	5,562,687	*bla*_PLA1a_, *fosA*	—
pGY22PK019_1	167,410	*tmexCD1*‐*toprJ1*, *bla*_DHA-1_, *aph(4)-Ia*, *aac(3)-IV*, *aadA1*, *aadA2*, *strA*/*B*, *qnrB4*, *cmlA1*, *sul1*, *sul3*	IncFIB
pGY22PK019_2	108,686	*bla*_TEM-1B_, *bla*_CTX-M-3_, *tet*(A), *aac(3)-IId*, *aac(6’)-Ib-cr*, *aph(3’)-Ia*, *strA*/*B*, *aadA16*, *qnrS1*, *floR*, *sul1*, *sul2*, *dfrA27*, *mph*(A), *arr-3*	IncQ1‐IncFII(K)‐IncF_repB(R1701)_
pGY22PK019_3	7631	—	Col
GY22PK020			
Chromosome	5,564,573	*bla*_PLA1a_, *fosA*	—
pGY22PK020_1	280,733	*bla*_DHA-1_, *aph(4)-Ia*, *aac(3)-IV*, *aadA1*, *aadA2*, *strA*/*B*, *qnrB4*, *cmlA1*, *sul1*, *sul3*	IncFIB/IncHI1B
pGY22PK020_2	167,401	*tmexCD1*‐*toprJ1*, *bla*_DHA-1_, *aph(4)-Ia*, *aac(3)-IV*, *aadA1*, *aadA2*, *strA*/*B*, *qnrB4*, *cmlA1*, *sul1*, *sul3*	IncFIB
pGY22PK020_3	108,693	*bla*_TEM-1B_, *bla*_CTX-M-3_, *tet*(A), *aac(3)-IId*, *aac(6’)-Ib-cr*, *aph(3’)-Ia*, *strA*/*B*, *aadA16*, *qnrS1*, *floR*, *sul1*, *sul2*, *dfrA27*, *mph*(A), *arr-3*	IncFII(K)
GY22PK021			
Chromosome	5,562,564	*bla*_PLA1a_, *fosA*	—
pGY22PK021_1	167,410	*tmexCD1*‐*toprJ1*, *bla*_DHA-1_, *aph(4)-Ia*, *aac(3)-IV*, *aadA1*, *aadA2*, *strA*/*B*, *qnrB4*, *cmlA1*, *sul1*, *sul3*	IncFIB
pGY22PK021_2	108,685	*bla*_TEM-1B_, *bla*_CTX-M-3_, *tet*(A), *aac(3)-IId*, *aac(6’)-Ib-cr*, *aph(3’)-Ia*, *strA*/*B*, *aadA16*, *qnrS1*, *floR*, *sul1*, *sul2*, *dfrA27*, *mph*(A), *arr-3*	IncQ1/IncFII(K)/IncF_repB(R1701)_
pGY22PK021_3	7631	—	Col
GY22PK022			
Chromosome	5,565,064	*bla*_PLA1a_, *fosA*	—
pGY22PK022_1	167,408	*tmexCD1*‐*toprJ1*, *bla*_DHA-1_, *aph(4)-Ia*, *aac(3)-IV*, *aadA1*, *aadA2*, *strA*/*B*, *qnrB4*, *cmlA1*, *sul1*, *sul3*	IncFIB
pGY22PK022_2	108,679	*bla*_TEM-1B_, *bla*_CTX-M-3_, *tet*(A), *aac(3)-IId*, *aac(6’)-Ib-cr*, *aph(3’)-Ia*, *strA*/*B*, *aadA16*, *qnrS1*, *floR*, *sul1*, *sul2*, *dfrA27*, *mph*(A), *arr-3*	IncQ1/IncFII(K)/IncF_repB(R1701)_
pGY22PK022_3	13,913	—	Col
GY22PK023			
Chromosome	5,562,668	*bla*_PLA1a_, *fosA*	—
pGY22PK023_1	167,405	*tmexCD1*‐*toprJ1*, *bla*_DHA-1_, *aph(4)-Ia*, *aac(3)-IV*, *aadA1*, *aadA2*, *strA*/*B*, *qnrB4*, *cmlA1*, *sul1*, *sul3*	IncFIB
pGY22PK023_2	99,043	*bla*_TEM-1B_, *bla*_CTX-M-3_, *tet*(A), *aac(6’)-Ib-cr*, *aadA16*, *qnrS1*, *floR*, *sul1*, *dfrA27*, *mph*(A), *arr-3*	IncFII(K)/IncF_repB(R1701)_
pGY22PK023_3	7631	—	Col

Virulence gene analysis based on VFDB revealed that the majority of virulence genes were chromosomally encoded. All isolates carried multiple determinants involved in bacterial adhesion, biofilm formation, and iron acquisition, which are essential for colonization and pathogenicity. Genes associated with fimbrial assembly, including *fimABCDEFGHIK* (type 1 fimbriae) and *mrkABCDFHIJ* (type 3 fimbriae), were identified in all strains, supporting their ability to adhere to host tissues and persist in hostile environments. Additionally, iron acquisition systems were consistently present. All strains carried the *iutA* gene encoding the aerobactin receptor, as well as genes involved in enterobactin synthesis and transport (*entABCDEFS*, *fepABCDG*). The yersiniabactin operon (f*yuA*, *irp1/2*, *ybtAEPQSTUX*) was also detected, suggesting the presence of multiple siderophore‐mediated iron uptake systems (Figure 1 and Supporting Informations 2: Table S2). These virulence factors likely enhance survival under iron‐limiting conditions and contribute to immune evasion and systemic dissemination.

### 3.3. Phylogenetic Analysis of *tmexCD1-toprJ1*‐Positive *R. ornithinolytica* Isolates

To explore the genetic diversity, phylogenetic analysis was conducted based on core‐genome SNPs, incorporating 18 *R. ornithinolytica* genomes from 11 countries available in the NCBI database. The strains clustered into four distinct clades. All six isolates from this study were grouped within the fourth clade, and showed the closet relationship to strain HCA1847, previously isolated from a wound infection in Ecuador (Figure 1). Pairwise SNP comparisons revealed very low genetic diversity among the six isolates, with only 13–32 SNP differences, suggesting recent clonal expansion or transmission (Supporting Informations 3: Table S3).

### 3.4. Genetic Environment of *tmexCD1-toprJ1*

Comparative plasmid analysis showed that the *tmexCD1-toprJ1*‐carrying plasmids recovered from the six *R. ornithinolytica* in this study were highly conserved, exhibiting 100% sequence coverage and over 99.99% nucleotide identity with one another. Further BLASTN comparison with plasmids available in the GenBank database showed that these plasmids shared more than 66% sequence coverage and over 98.84% nucleotide identity with five reference plasmids. These included three plasmids from *K. pneumoniae* isolates obtained from patient blood samples in South Korea: pC17KP0040‐1 (CP052393.1), pE16KP0102‐2 (CP052311.1), and pE16KP0224‐1 (CP052281.1); and two plasmids also derived from *K. pneumoniae* strains recovered from animal feces in China: pYZEF2‐2C‐2 (CP128486.1) and pYZE1‐3D‐2(CP129187.1) (Figure 2A). Plasmid replicon typing indicated that the *tmexCD1-toprJ1*‐carrying plasmids from all six isolates belonged to the IncFIB incompatibility group, which differs from the IncFIA plasmid pHNAH8I‐1, the first *tmexCD1-toprJ1*‐positive plasmid identified, which was isolated from a *K. pneumoniae* strain recovered from chicken cloaca swabs.

Figure 2Comparative genomic analysis of *tmexCD1-toprJ1*‐harboring plasmids. (A) Circular comparison of representative *tmexCD1-toprJ1*‐carrying plasmids generated using BRIG. Circular alignment of eleven complete *tmexCD1-toprJ1*‐positive plasmids, including five plasmids from this study and six reference plasmids retrieved from GenBank. Circles from the inside to outside indicate plasmids pC17KP0040‐1 (CP052393.1), pE16KP0102‐2 (CP052311.1), pE16KP0224‐1 (CP052281.1), pYZEF2‐2C‐2 (LC765517.1), pYZE1‐3D‐2 (CP129187.1), pGY22PK023_1 (this study), pGY22PK022_1 (this study), pGY22PK021_1 (this study), pGY22PK020_2 (this study), pGY22PK019_1 (this study), and pGY22PK011_1 (this study). (B) Linear alignment of resistance regions among *tmexCD1-toprJ1*‐carrying plasmids visualized using Easyfig. Linear comparison of three *tmexCD1-toprJ1*‐positive plasmids from this study with three representative reference plasmids. Genes are shown as arrows indicating transcriptional direction, with color coding based on gene function. Regions with ≥ 90.0% nucleotide identity are shaded in gray. Truncated genes are indicated by the symbol *Δ*.

**Figure:**
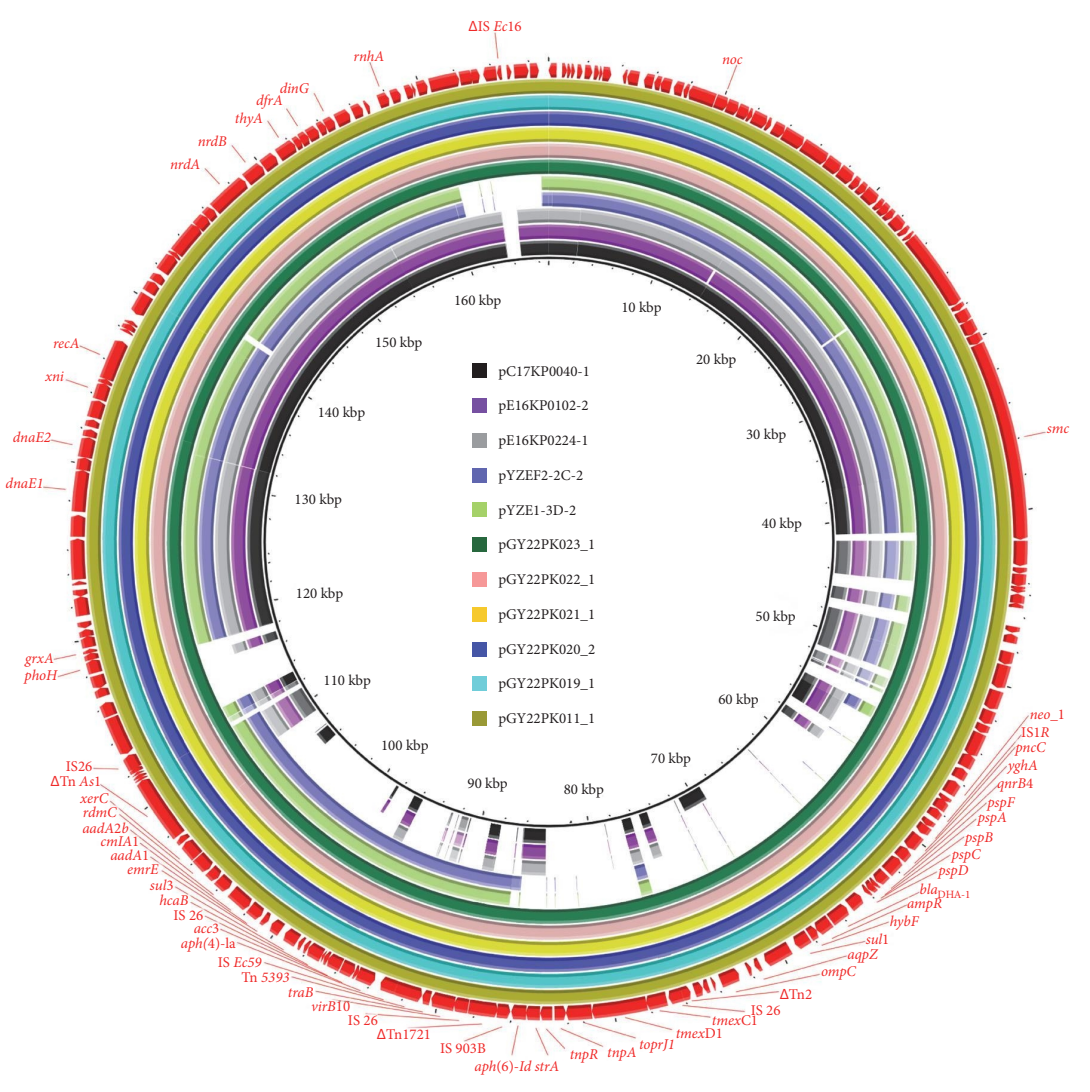
(A)

**Figure:**
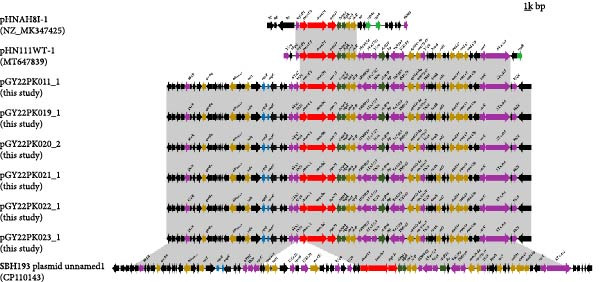
(B)

Notably, analysis of the genetic context surrounding the *tmexCD1-toprJ1* gene cluster in the six plasmids revealed the presence of an incomplete transposase gene *Tn2* and a complete insertion sequence *IS26* located upstream of the resistance cluster. These elements appear to have replaced the *tnfxB1* regulatory gene found in earlier reported plasmids such as pHNAH8I‐1 and pHN111WT‐1 (Figure 2B), indicating structural rearrangements that may have facilitated plasmid evolution and mobility.

### 3.5. Virulence Evaluation in the *G. mellonella* Model

**Figure 3:**
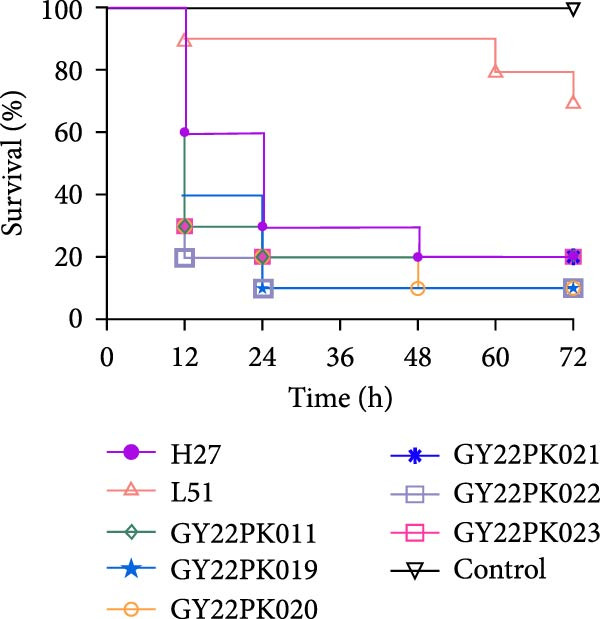
Virulence assessment of *tmexCD1-toprJ1*‐positive *R. ornithinolytica* isolates using the *G. mellonella* infection model. Survival curves of *G. mellonella* larvae (*n* = 10 per group) infected with representative isolates from this study (GY22PK011‐GY22PK023), compared with the high‐virulence reference strain H27 and the low‐virulence reference strain L51. Larvae were monitored for 72 h postinfection. Each infection assay was performed independently three times, and the survival curves shown represent data from one representative experiment, as consistent trends were observed among replicates. Lower survival rates in larvae infected with the test isolates indicate higher virulence compared with the reference strains.

The virulence of six *tmexCD1-toprJ1*‐positive *R. ornithinolytica* isolates was evaluated using the *G. mellonella* larvae infection model. At 12 h postinfection, the survival rate of larvae infected with strains GY22PK011, GY22PK020, GY22PK021, and GY22PK023 were 30%, while GY22PK019 showed a slightly higher survival rate of 40% (Figure 3). The lowest survival rate at this time point was observed for GY22PK022 (20%). In comparison, the survival rates for the low‐virulence *K. pneumoniae* strain L51 and hypervirulent strain H27 were 90% and 60%, respectively. By 72 h postinfection, survival rates for GY22PK011, GY22PK021, and GY22PK023 had declined to 20%, comparable to the level observed for strain H27 (Figure 3). Notably, survival for GY22PK019, GY22PK020, and GY22PK022 further dropped to 10%, indicating more pronounced virulence. All six *R. ornithinolytica* isolates were associated with higher mortality than the low‐virulence *K. pneumoniae* strain L51, and no mortality was observed in the PBS‐injected control group. These results suggest that *tmexCD1-toprJ1*‐positive *R. ornithinolytica* isolates possess considerable virulence potential in vivo, with some strains exhibiting lethality levels approaching those of hypervirulent *K. pneumoniae*, in addition to their multidrug resistance profiles.

## 4. Discussion

Efflux pumps play a critical role in antimicrobial resistance by actively extruding antibiotics from bacterial cells, thereby reducing intracellular drug concentrations. *MexCD-OprJ* is a chromosomally encoded RND‐type efflux pump in *Pseudomonas* spp., contributing to intrinsic resistance against multiple antibiotics [6, 60, 61]. In 2020, a plasmid‐mediated RND‐type efflux pump gene cluster, *tmexCD1-toprJ1*, was first identified in *K. pneumoniae* of animal origin. Since then, *K. pneumoniae* has remained the predominant host, although sporadic detections have been reported in *Raoultella planticola* in 2022 and *Pseudomonas stutzeri* in 2023 [30, 62]. However, the complete host range of *tmexCD-toprJ* remains largely undefined.

In this study, *tmexCD1-toprJ1* was detected in six *R. ornithinolytica* isolates obtained from a swine farm in Jiangsu Province, with an isolation rate of 4.76% (6/126). These findings expand the known ecological distribution of the *tmexCD1-toprJ1* gene cluster by identifying it in *R. ornithinolytica* recovered from livestock‐associated drinking water systems. Previous reports of *tmexCD-toprJ* variants have primarily focused on clinical isolates of *K. pneumoniae* and related Enterobacteriaceae, whereas our results demonstrate that this resistance determinant is also present in environmental niches connected to food animal production. The detection of *tmexCD1-toprJ1* in drinking water samples suggests that farm water systems may serve as previously unrecognized reservoirs and potential transmission media, either through contamination by animal feces or persistence within aquatic microbial communities. Five of six *tmexCD1-toprJ1*‐positive isolates were obtained from drinking water rather than directly from pigs, reinforcing the hypothesis that farm water may represent a critical reservoir. Given that water systems connect multiple ecological compartments, including animals, the farm environment, and potentially humans, our results underscore the importance of monitoring farm‐associated water as a critical component of antimicrobial resistance surveillance under the One Health perspective.

Despite this, tigecycline is rarely used in livestock, but the widespread and prolonged use of tetracyclines as growth promoters and prophylactic agents may exert selective pressure that indirectly facilitates the persistence of *tmexCD1-toprJ1* and its variants [6]. Swine manure has been recognized as a rich source of ARGs, capable of contaminating farm environments and potentially contributing to horizontal gene transfer to farm workers or surrounding ecosystems [63, 64].Given that *R. ornithinolytica* is frequently recovered from aquatic sources and has been implicated as a reservoir for ARGs [14, 15, 65], its high recovery rate from drinking water samples in this study suggests a potential environmental transmission pathway for *tmexCD1-toprJ1*. These findings underscore the urgent need for enhanced ARG surveillance in livestock settings and stricter regulation of antibiotic use to mitigate resistance spread.

Plasmids are central to the global dissemination of resistance determinants such as *tmexCD1-toprJ1*. Conjugative plasmids often encoded key modules required for horizontal transfer, including an origin of transfer (oriT), a relaxase, a type IV coupling protein (T4CP), and a type IV secretion system (T4SS) [66, 67]. In this study, conjugation and electroporation experiments failed to transfer the *tmexCD1-toprJ1*‐positive plasmids into *E. coli* C600 or DH5*α*. In silico analysis using the Galaxy platform and oriTDB confirmed the absence of classical conjugation‐associated modules, indicating that the IncFIB plasmids carrying *tmexCD1-toprJ1* are likely nonconjugative and nonmobilizable.

Nevertheless, the identification of *IS26* elements flanking the gene cluster suggests potential for mobilization. *IS26* has been widely implicated in mediating the rearrangement and horizontal transfer of resistance determinants among Enterobacteriaceae. It is possible that the mobilization of *tmexCD1-toprJ1* requires specific conditions, alternative recipient strains, or additional helper plasmids, which were not captured in our experimental design. Therefore, while our current data indicate low transferability, the presence of *IS26* warrants further investigation into the potential mobility of these plasmids.

Comparative analysis of *tmexCD1-toprJ1*‐carrying plasmids revealed structural variations, including the presence of IS*26* and the complete absence of *tnfxB1*. *tnfxB1* encodes a TetR‐type transcriptional regulator that normally represses the *tmexCD-toprJ* efflux pump cluster. Recent studies have shown that inactivation or loss of *tnfxB1* can mediate an 8‐ to 16‐ fold increase in tigecycline MICs by derepressing efflux pump expression [68, 69]. In our isolates, the absence of *tnfxB1* may therefore abolish transcriptional repression and contribute to elevated tigecycline resistance, although this requires experimental validation. In addition, *IS26*‐mediated rearrangements may facilitate plasmid evolution and contribute to the dissemination of resistance genes. Together, these genomic features provide mechanistic clues to how plasmid architecture might affect the persistence and expression of *tmexCD1-toprJ1*. Future work incorporating transcriptomic or promoter activity analyses will be valuable in elucidating the impact of these rearrangements.

This arrangement is particularly significant, as previous studies have shown that pairs of *IS26* can mediate the formation of circular intermediates, facilitating gene mobilization through homologous recombination [70, 71], highlighting that horizontal gene transfer can occur independently of conjugation machinery. More importantly, Wang et al. [72] provided direct in vitro and in vivo experimental validation of *IS26*‐driven transposition in *Salmonella enterica*. They showed that *IS26* mediated replicative, intramolecular (cis and trans), and composite transpositions to restructure resistance regions (RRs), generate new lineages, and even enable host adaptation. In their model, four *IS26* copies flanking RR3 facilitated multiple deletion, inversion, and recombination events, giving rise to stable new serovar structures with enhanced fitness.Their findings confirm that *IS26* elements alone are sufficient to drive complex genomic remodeling under selective pressures. Therefore, while we did not observe direct mobilization experimentally, the presence of *IS26* in dual orientation flanking *tmexCD1-toprJ1* strongly suggests that this resistance cluster could act as a “latent mobile unit”. Therefore, while direct conjugation may not be possible, the risk of future mobilization via *IS26*‐mediated mechanisms cannot be excluded. This remains a serious concern, as the potential integration of *tmexCD1-toprJ1* into other mobile elements or chromosomes could facilitate its further dissemination, potentially resulting in untreatable infections and significant public health implications.

In addition to resistance determinants, all six isolates carried multiple virulence‐associated genes, including *mrkABCDFHIJ*, *fimABCDEFGHIK*, *iutA*, *entABCDEFS*, *fepABCDG*, *fyuA*, *irp1/2*, and *ybtAEPQSTUX*, which contribute to bacterial adherence, biofilm formation and iron acquisition [30]. However, classical hypervirulence markers such as *peg-344*, *iroB*, *iucA*, *rmpA*, and *rmpA2* were not detected [73].

The virulence of the isolates was evaluated using the *G. mellonella* infection model, where moderate to high pathogenicity was observed. However, this result should be interpreted with caution, as only six isolates were tested and no classical hypervirulence‐associated genes were detected in their genomes. We therefore consider the outcome as an indication of potential pathogenicity rather than definitive evidence of high virulence. Additional experiments with larger isolate collections and complementary animal models will be required to better characterize the virulence potential of *tmexCD1-toprJ1*‐carrying *R. ornithinolytica*.

Although *R. ornithinolytica* is predominantly associated with environmental reservoirs such as soil and water [14, 15, 65, 74, 75], its increasing recovery from clinical specimens [15, 76, 77] and animal slaughterhouse environments [29] suggests its potential role as an emerging opportunistic pathogen. The rising prevalence of tigecycline resistance underscores the urgent need for comprehensive surveillance programs to monitor the emergence and dissemination of MDR pathogens. Safeguarding the clinical efficacy of tigecycline and other last‐resort antibiotics requires a multifaceted strategy, including strict antimicrobial stewardship, improved infection control practices, and the development of alternative therapeutic approaches. In light of the present findings, sustained research efforts are essential to elucidate the ecological and clinical significance of *tmexCD1-toprJ1* in *R. ornithinolytica* and to guide the implementation of effective interventions aimed at limiting the spread of MDR strains and protecting public health.

Taken together, our findings expand the known ecological distribution of *tmexCD1-toprJ1* by demonstrating its presence in *R. ornithinolytica* from livestock‐associated water systems. This highlights the role of agricultural environments, particularly farm drinking water, as potential reservoirs and transmission interfaces for tigecycline resistance. The coexistence of multidrug resistance and moderate pathogenic potential in these isolates underscores the importance of enhanced surveillance and containment strategies. While our study is limited by the relatively small number of isolates and the fact that all belonged to the same species, the results nonetheless provide new insights into the ecology and possible transmission pathways of *tmexCD-toprJ*. These findings lay a foundation for future One Health‐oriented, large‐scale surveillance, and mechanistic investigations aimed at mitigating the dissemination of *tmexCD-toprJ*.

## 5. Conclusions

This study identified the mobile tigecycline resistance gene cluster *tmexCD1-toprJ1* in *R. ornithinolytica* isolates recovered from a swine farm in China. Genomic analysis revealed a high degree of homogeneity among the isolates, suggesting possible clonal dissemination. Although plasmid transfer was not observed under laboratory conditions, the presence of mobile genetic elements indicates a potential for horizontal gene transfer in natural settings. In addition to multidrug resistance, the isolates exhibited notable virulence traits, raising concerns about their pathogenic potential and implications for public health. These findings highlight the importance of strengthened antimicrobial resistance surveillance in livestock environments to monitor and limit the dissemination of resistance determinants such as *tmexCD1-toprJ1*.

## Ethics Statement

The authors have nothing to report.

## Conflicts of Interest

The authors declare no conflicts of interest.

## Author Contributions

**Hanyun Wang:** investigation, methodology, formal analysis, writing – original draft. **Yujing Zhang:** investigation, methodology, validation, visualization, writing – original draft. **Zhenyu Wang:** visualization, data curation. **Yawen Xu:** methodology, resources. **Le Zhou:** methodology, resources. **Jing Wang:** conceptualization, methodology. **Xinan Jiao:** conceptualization, funding acquisition, supervision. **Lin Sun:** conceptualization, project administration, supervision, writing – review and editing.

## Funding

This work was supported by the fifth phase of the “333 Project” scientific research project in Jiangsu Province (Grant BRA2020002), the Priority Academic Program Development of Jiangsu Higher Education Institution (PAPD), the Postgraduate Research & Practice Innovation Program of Jiangsu Province (SJCX22_1768), and the Medical Research Project of Yangzhou Commission of Health (2023‐2‐33).

## Supporting Information

Additional supporting information can be found online in the Supporting Information section.

## Supporting information


**Supporting Information 1** Table S1. *tmexCD-toprJ*‐positive *Raoultella ornithinolytica* isolates obtained from a swine farm.

**Supporting Information 2** Table S2. Presence or absence of virulence genes from VFDB in *Raoultella ornithinolytica* strains in this study.

**Supporting Information 3** Table S3. Pairwise distance matrices of cgSNPs for *tmexCD1-toprJ1*‐positive *Raoultella ornithinolytica* isolates in this study.

## Data Availability

All genome sequences generated in this study have been deposited in the NCBI GenBank under BioProject Accession Number PRJNA921819.
