# Supplementary material for: Emergence of Mobilized Tigecycline Resistance Gene Cluster tmexCD1-toprJ1 in Raoultella ornithinolytica From a Swine Farm, China
Source: Transbound Emerg Dis. 2025 Dec 18;2025:6690944. doi: 10.1155/tbed/6690944 (PMC12714096; doi:10.1155/tbed/6690944)
Supplement: Supplementary file 1 — Supporting Information 1 Table S1. tmexCD-toprJ‐positive Raoultella ornithinolytica isolates obtained from a swine farm. [file TBED-2025-6690944-s001.docx]

**Table S1. *tmexCD-toprJ*-positive *Raoultella ornithinolytica* isolates obtained from a swine farm.**

| **Types of samples** | **No. of samples** | **No. of**  ***tmexCD-toprJ*-positive isolates (%)** |
| --- | --- | --- |
| swine feces | 84 | 1（1.19%） |
| floor wipe | 31 | 0 |
| drinking water | 5 | 5（100%） |
| swine feed | 3 | 0 |
| soil | 3 | 0 |
| total | 126 | 6（4.76%） |
